# On Horny Growth of the Penis, with Exhibition of a Remarkable Case

**Published:** 1887-07

**Authors:** 


ON HORNY GROWTH OF THE PENIS, WITH EXHIBITION OF A REMARKABLE CASE.—Dr. J. H. Brinton, of Philadelphia, read a paper on the above subject before the recent meeting of the American Association of Genito-Urinary Surgeons, exhibiting a specimen and referring to those on record. His specimen was from a man on whose penis a horn had existed more than four years, having started from a wart. The wart had itched occasionally, and the patient had scratched it for this reason. Gradually it turned into a horny substance. It caused no trouble, except mechanical interference with coition. The horn sprang from the base of the glans, at the coronary border, and was attached to both the glans and prepuce; it was one and seven-eighths inches long, one and three-eighths inches in circumference; it was curved forward. A peculiar feature in this particular case was the fact that the horny plate surrounding the meatus almost occluded the meatus, so that the urine passed only in drops. The urethra behind the horny plate was not contracted. The horn was cut off, and the man left the hospital after about three weeks.

The rarity of horny growths upon the penis was somewhat remarkable. He was surprised to find only fourteen cases recorded in English, German and French literature. A few more cases had been vaguely alluded to. They occurred either as well-marked projecting horns, or as rough, flat, horny plates occupying the glans penis; they were sometimes multiple. The longest on record was three inches.—*Maryland Medical Journal.*

